# The Short-Term Effects of *Pistacia Lentiscus* Oil and Sesame Oil on Liver and Kidney Pathology of Rats and Human Cancer Cell Lines

**DOI:** 10.31661/gmj.v9i0.2001

**Published:** 2020-12-29

**Authors:** Maryam Ostovan, Mohammad Hossein Anbardar, Hajar Khazraei, Seyyed Mohammad bagher Fazljou, Zahra Khodabandeh, Seyedeh Azra Shamsdin, Mostafa Araj Khodaei, Mohammadali Torbati

**Affiliations:** ^1^Department of Persian Medicine, School of Traditional Medicine, Tabriz University of Medical sciences, Tabriz, Iran; ^2^Department of Pathology, Medicine Faculty, Shiraz University of Medical Sciences, Shiraz, Iran; ^3^Colorectal Research Center, Shiraz University of Medical Sciences, Shiraz, Iran; ^4^Stem cell Technology Research Center, Khalili Street, Research Tower, Shiraz University of Medical Science, Shiraz, Iran; ^5^Gastroenterohepatology Research Center, Shiraz University of Medical Sciences, Shiraz, Iran; ^6^Department of Traditional Pharmacy, School of Traditional medicine, Tabriz University of Medical Sciences, Tabriz, Iran

**Keywords:** Mastic, Rats, Sesame Oil, Liver, Kidney

## Abstract

**Background:**

Vegetable oils recently have been evaluated in many tissues. *Pistacia lentiscus* (mastic) of the Anacardiaceae family and *Sesamum indicum* (sesame) of the Pedaliaceae family are conventionally used in the management of gastrointestinal, lung, and skin illnesses. This assay attempts to determine if the oral usage of mastic and sesame oils has any short-term toxic effects *in vivo* on the rat and evaluate the human anticancer effect *in vitro*.

**Materials and Methods:**

Twenty-one male Sprague-Dewley rats were assigned to three groups randomly: (A) control, (B) mastic oil (400 mg/kg), and (C) sesame oil (2cc/kg). The effects of these oils were investigated by determining histopathological and stereological parameters after six days, and the anticancer effects were evaluated on SW48, HepG2 human cell lines.

**Results:**

A mild chronic interstitial inflammation was seen in just one kidney of mastic oil group (B) and the other ones were normal. In the sesame oil group (C), mild chronic interstitial inflammation was seen in six kidneys. In the liver samples of both groups, there were no specific pathological findings. Different concentrations of mastic oil (0.1%-5%) reduced the cell viability of SW48, HepG2, HEK293t, and human fat cells.

**Conclusion:**

Mastic and sesame oils have some side-effects on the kidney and might not be safe at high doses in rats. Sesame oil did not have any toxic effect on HepG2 and HEK293t human cancer cells. Mastic oil treatment has inhibited specific SW48 cells, so this oil seems to be a good adjuvant to chemotherapy in colon treatments.

## Introduction

The *Pistacia lentiscus* (var.chia) tree is from *Anacardiaceae* family with a height of 4 meters produces reddish or yellowish natural resin gum (mastic), which grows all over the Mediterranean region [1]. Some studies on the essential oil of mastic gum reported the anti-inflammatory, anticancer effects, antibacterial, antifungal, and antiviral activities [2-4]. Mastic is consumed as a traditional treatment in Mediterranean region, especially Iran [4]. *P.lentiscus* was used in the gastric ulcer treatment [5-6] and healing burns [7]. In one study, *P.lentiscus* fatty oil consumed by rabbits rectally significantly reduced the alanine and aspartate transaminase in the plasma. They did not report any significant toxicity on the liver and renal functions after using *P.lentiscus* [8]. In one study, the effect of mastic resin of *Pistacia atlantica***was in**vestigated in bile duct cancer, pancreatic carcinoma, gastric adenocarcinoma, and colonic adenocarcinoma, reporting the suppression of cell proliferation [9]. Sesame oil demonstrated the anti-inflammatory and repair effect but might produce an allergic reaction [10]. It has antihypertensive, neuroprotective, and anticancer effects [11]. In one animal study, sesame oil showed a hepatoprotective effect against oxidative liver injury, and reduced the lipid peroxidation by elevated serum liver antioxidant enzymes [11]. In traditional medicine, mastic (with sesame oil) is usually used for therapeutic aim or as a flavoring agent without any reported toxicity [12]. According to the above-mentioned points and the commonly usage of mastic in gastrointestinal problems, we want to use the mastic and sesame oil orally for rats and measure if it shows any short-term toxic effects. The kidney and liver are two major organs with vital roles in human physiology like synthesis of useful components and metabolism and excretion of macromolecules. Some natural products cause liver or kidney damages and abnormalities.

To the best of our knowledge, the toxicity of mastic and sesame oils in normal tissues of the liver or kidney has not been reported so far, and the effects of mastic and sesame oils have not been evaluated in colon cancer cell line.Our study aimed to investigate the short-term effects of mastic and sesame oil on the kidney and liver in rats in vivo ** s**ix days after oral administration and in vitro cell viability assessment on human colon cancer cell line.

## Materials and Methods

 The present study was approved by ethics committee of Shiraz University of Medical Sciences (IR.SUMS.REC.1399.813).

###  Chemicals

 Formalin, acetic acid, DMSO and diethyl ether were purchased from Merck Co.(Darmstadt, Germany). Sesame oil was purchased from Moosavi Co. (Kazeroon, Iran). Trypsin 0.25% (Shellmax, China) was used for the cell culture.

###  Mastic Oil Preparation

Mastic (the resin from *P.lentiscus*) was purchased from herbal shop (Shiraz, Fars, Iran). It was identified in Department of Pharmacognosy (Shiraz University of Medical Sciences). 40 g of mastic was dissolved in 200cc sesame oil (in boiling water). Then, the solution was mixed to become uniform [13]. The dose of mastic oil (400 mg/kg) was chosen based on traditional references [12,13].

###  Animals

 A total of 21 male Sprague-Dewley rats (6-8 week old) weighing 200-250 g were purchased from the animal lab (Shiraz University of Medical Sciences, Shiraz, Iran). All the rats were kept in temperature and humidity-controlled rooms (20-23 °C, 50-60 %) in 12-h light/12-h dark cycle and free access to standard laboratory chow with tap water ad libitum in stainless steel cages according to the local guidelines in Shiraz University of Medical Sciences.

###  Experimental Design

 The following groups were designed and the rats were allocated into three groups: Sham group (n=7): (A) normal saline (2 ml/kg/day); and Treatment groups (n=7): (B) mastic oil (400 mg/kg/day), and (C) sesame oil (2 ml/kg/day). Medication was used daily via oral gavage for six consecutive days.

###  Histopathological and Histomorphometric Assessment

 The animals were sacrificed on the seventh day and their kidneys and livers were excised. The tissue samples fixed in formalin were dried, embedded in paraffin, and then cut into slices of 5 μm thickness. Then, the slices were dyed by hematoxylin and eosin method. The renal cortex part and the renal medulla, proximal and distal convoluted tubule diameters were assayed and glomerulus diameter and capsule’s thickness, Henle’s loop, and collecting ducts were measured.

###  Impact of Mastic and Sesame Oil on Human Cellular Viability in Vitro 

 Human colon cancer cells SW48, liver cancer cells HepG2, embryonic kidney cells HEK293t, and human fat cells were kept in DMEM with penicillin 50U/ml and streptomycin 50µg/ml with 10% fetal bovine serum (Shellmax, China). 5000 cells/well were incubated in a 96-well plate for 24 h. Then, cells were treated for another 48 h with different dilutions of mastic and sesame oil (0.1%, 0.5%, 1%, 5 % with 0.1% DMSO), in triplicate. 5-FU (Ebefluoro, Iran), was used as the control treatment, and well dissolved in the DMEM media. The cell viability was determined after treatment of mastic and sesame oil by MTT (3-(4, 5-dimethylthiazol-2-yl)-2, 5-diphenyltetrazolium bromide, Sigma, USA) assay in 570nm. Data is presented by percentage of cell viability (%) in comparison with the untreated cells (the viability 100%).

###  Statistical Analysis 

 The in vitro experiments were performed in the sets of triplicate for 48hr and statistical data were presented by mean value of ± standard deviation. All the experimental data were analyzed by the unpaired student t-test, using SPSS (version 18; SPSS Inc. Chicago, USA). The P<0.05 was considered as significant value.

## Results

###  Effect of Mastic and Sesame Oil on Histopathological Features and Histomorphometric Measurements in Rats

**Figure 1:**
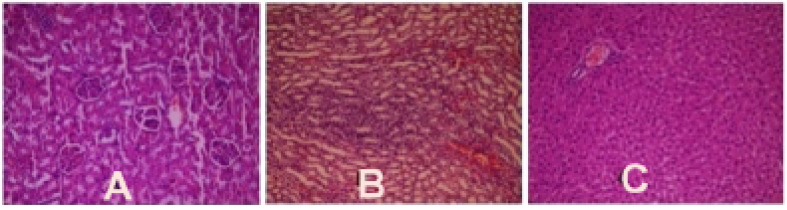
Mastic group. **A:** Section from kidney shows no pathologic change. **B: **Section from kidney shows focal interstitial inflammation. **C: **Section from liver shows no pathologic change. (H&E, ×200)

**Figure 2:**
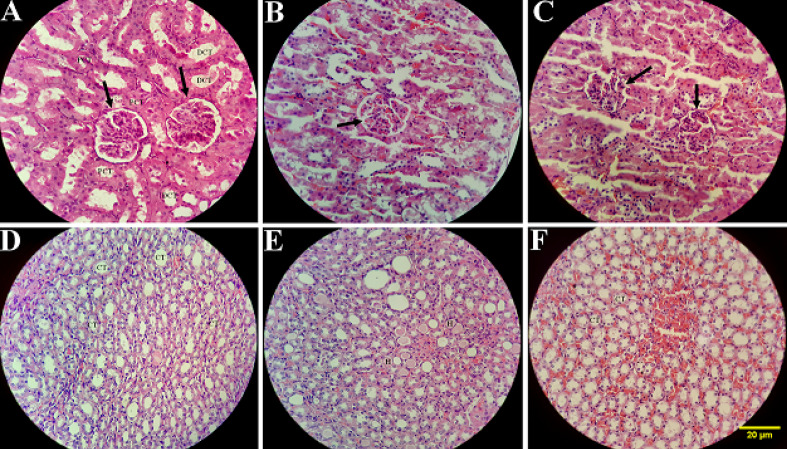
A comparison of the renal tissue in experimental groups. ( **A, D**) The normal tissue. ( **B, E**) The renal tissue after the administration of sesame. ( **C, F**) the renal tissue after the administration of mastic. (H and E, ×400). images A, B, and C show the renal cortex part and images D, E, and F of the renal medulla. The arrow in A, B, and C indicate glomerulus diameter and capsule’s thickness. **PCT: **proximal convoluted tubule; **DCT:** Distal convoluted tubule diameter; **H: **Henle’s loop, and **CT: **Collecting ducts.

**Figure 3:**
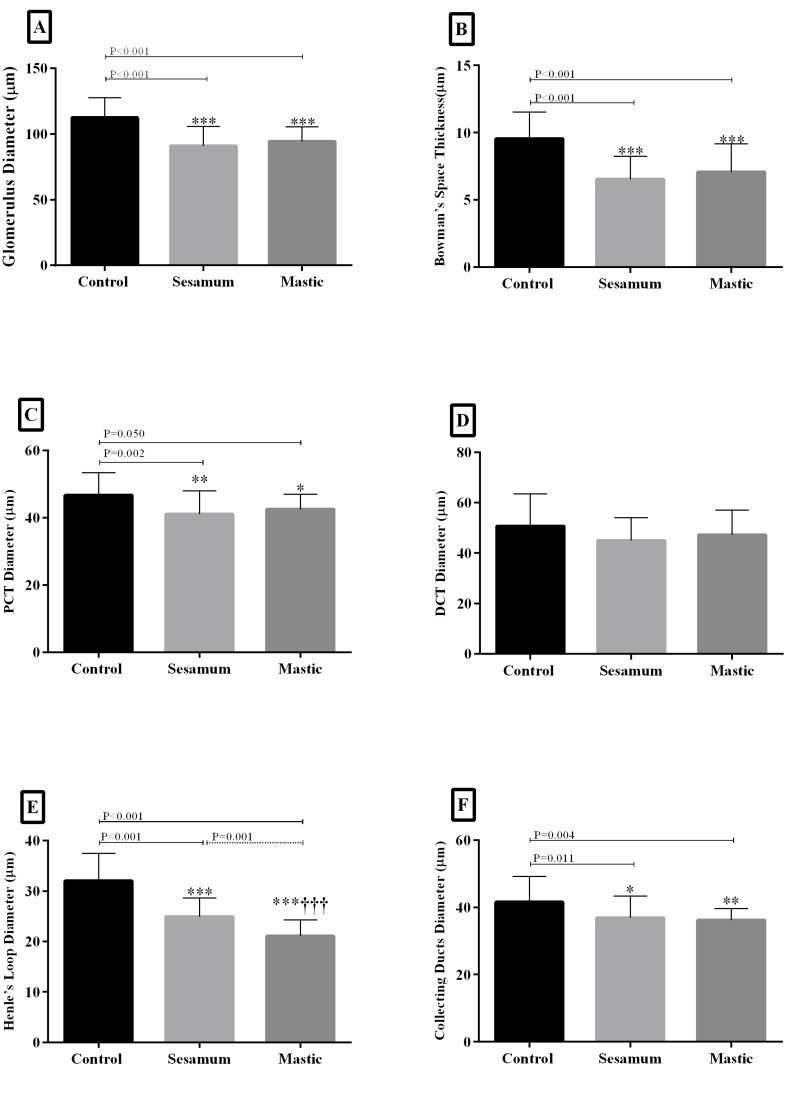
The results of histomorphometric parameters in different groups. (Mean ± SD). ** PCT:** proximal convoluted tubule diameter; **DCT: **Distal convoluted tubule diameter.
 *P<0.05, Control group vs. sesame and mastic groups. †p<0.05, sesame vs. mastic group.

 The group A showed no histological damage; hence, the mucosa had an intact epithelium. In one kidney (group B), a mild chronic interstitial inflammation was detected (Figure-1A), and the group C, showed mild chronic interstitial inflammation in six kidneys (Figure-1 B). There were no specific pathological findings in the liver samples of the two treated groups B and C (Figure-1 C). All the experiments were repeated three times in the kidney of all rats (Figure-2 and 3). The renal cortex and image of the renal medulla demonstrated that the glomerulus diameter and capsule’s thickness in two groups B and C were reduced significantly (P<0.001). The proximal convoluted tubule decreased in both groups, but the distal convoluted tubule diameter did not significantly change. Henle’s loop and collecting ducts significantly decreased in both groups (Figure-3). In the group B, the Henle’s loop diameter was significantly different with the group C (P=0.001).

###  Effect of Mastic and Sesame Oil on Human Cellular Viability

 All the experiments were repeated three times. Human cancer cells SW48, HepG2, HEK293t and human fat cells were used in the MTT test. In Table-1, mastic and sesame oil dilutions (0.1%-5%) decreased the cellular viability over 48 hours, but it was higher in comparison with 5-FU as a control drug for cancer treatment.

## Discussion

We treated the rats orally with mastic and sesame oil for 6 consecutive days to show if these oils have any short-term toxic effects. In addition, cancer cells were incubated for 48 h with these oils to measure the cell viability. In our results, the liver samples had no specific pathological findings, but there was mild chronic interstitial inflammation in the kidney of mastic and sesame oil groups. Results in the renal cortex and medulla demonstrated that the glomerulus diameter, capsule’s thickness, Henle’s loop, and collecting ducts diameters decreased in both groups significantly. In another study, fatty oil of *P.lentiscus* was rectally administered for white male New Zealand rabbits, and the functions of liver and kidney were normal [8]. A chronic investigation demonstrated that mastic (high dose) increased the liver weights after 13 weeks [14]; however, we did not detect any damage in the rat’s liver, while other studies showed hepatoprotective effects of mastic oil in rats exposed to CCl4 [15] and intoxicated liver of rabbits by mercury [16]. In one animal study, hepatotoxicity that induced by lead acetate and treatment of sesame oil (5 ml/kg orally) after 30 days showed hepatoprotective effect. The structural changes in the liver significantly were improved by sesame oil. The alanine, aspartate aminotransferase, alkaline phosphatase and γ-glutamyltransferase activities were reduced, and total proteins and albumin were elevated [17]. Also, in a study by Hsu *et al.*, sesame oil potently attenuated damage of kidney and liver that induced by cisplatin through inhibiting the lipid peroxidation [18]. In an animal study by Hassanzade Taheri *et al*., using sesame oil increased the kidney and cortical volume and showed renal fibrosis. They suggested that renal deformities, such as fibrosis, dilatation, and tubular defects might be caused by high concentration of sesame oil. Hassanzade-Taheri *et al*. showed that sesame oil significantly increased the liver weight in rats [19]. On the other hand, in a study by Soleiman *et al*., sesame oil had renal and hepatoprotective effect against cypermethrin-induced degenerative and pathological changes [20]. In the present study, mastic oil dilutions (0.1-5%) decreased the cellular viability of SW48 and fat cells over 48 hours in a dose-dependent manner. Also, the oils showed some degree of cellular toxicity on human fat cells. These oils had no effects on HepG2 and HEK293t cells. Mastic showed the selective cytotoxicity against SW48 cells. The chemotherapeutic agent, 5-FU (100 µM), reduced cell viability to 70–85% of colon cancer (Caco-2) cells [21], while it reduced cell viability to 20.7% of SW48 cells in our study.

In one study, *P.Lentiscus* oil had no damage on the gastrointestinal mucosa and no alteration in cell proliferation [22]. Mastic oil in our study is completely different from *P.lentiscus* oil in their study [22]. We used mastic gum in sesame oil solution, but they used P.lentiscus fruit extract of essential oil. Mastic was effective on gastro-duodenal ulcer treatment [6]. In another study, mastic showed a potential role in the preneoplastic lesions formation in the rat liver [23]. On the other hand, our treatment of mastic oil showed inhibitory effects on the cell lines. Thus, the current data confirm the effect of mastic as a promising anti-neoplastic adjunct to cancer treatment. Future in vitro studies should investigate mastic and sesame oil on cell invasion, proliferation and growth analysis to identify the phenotypic changes of the colon cancer cells after mastic treatment. Further studies are warranted to identify the active compound (s), which selectively inhibit (s) the cell violability in colon cancer and fat cells. Also, it is recommended that some studies should be performed to find mastic and sesame oil pharmacological mechanism of action in vitro and in vivo.

## Conclusion

 Mastic and sesame oil have some side effects on the kidney and might not be safe at high doses in rats. These oils did not show any effects on HepG2 and HEK293t human cells. Mastic oil treatment has inhibited specific colon cancer cells, but due to its inhibitory effect on cell proliferation, this oil might be a good candidate for anti-tumour effect used in colon treatments.

## Acknowledgement

 This study was related to the thesis of Maryam Ostovan (no. 30), Tabriz University of Medical Sciences, and Tabriz, Iran. The authors wish to thank the Research Consultation Center (RCC) of Shiraz University of Medical Sciences for editing this manuscript. We thank from Farhad Kohpeyma for his technical assistance.

## Conflict of Interest

 The authors declare no conflict of interests.

**Table 1:** Cell Viability (% of control) Was Measured Using the MTT Test after Exposure of the SW48, Fat, HepG2, and Hek293t Cells to Mastic Oil, Sesame Oil, and 5-fluorouracil (5-FU) for 48 h.

**Cell lines**	**Treatments **	**Dilutions (%) /concentration (µg/ml)**	**Cell viability (%) ( Mean±SD )**	**P-value **
**SW48**	Mastic oil	5	50.0±15.1	0.024*
		1	60±45.4	0.322
		0.5	68.1±38.0	0.726
		0.1	77.8±29.5	0.341
		0.05	83.1±43.8	0.566
	Sesame oil	5	61.8±58.3	0.946
		1	87.9±64.0	0.581
		0.5	100±5.9	0.067
		0.1	100±27.2	0.132
	5-FU	250µg/ml	15.2±6.9	0.034*
		100µg/ml	20.7±2.4	0.02*
		10µg/ml	29.1±3.4	0.082
		1µg/ml	73.7±36.5	0.582
**Fat cell Human**	Mastic oil	5	44.3±13.6	0.522
		1	74±19.7	0.351
		0.5	77.1±67.6	0.126
		0.1	86.3±20	0.176
		0.05	95.1±34	0.271
	Sesame oil	5	48.7±22.9	0.042*
		1	66.1±29.7	0.102
		0.5	83.4±17.7	0.678
		0.1	100±24.1	0.317
	5-FU	250µg/ml	23.3±5.2	0.017*
		100µg/ml	67±28.1	0.330
		10µg/ml	69.7±24	0.128
		1µg/ml	80.6±10.2	0.191
**HepG2**	Mastic oil	0.5	100	1.00
		0.05	100	1.00
		0.005	79±10.3	0.654
	Sesame oil	0.5	100	1.00
		0.05	100	1.00
		0.005	100	1.00
	5-FU	250µg/ml	20.2±2.4	0.014*
		100µg/ml	25.6±7.9	0.032*
		10µg/ml	35±9.3	0.076
		1µg/ml	56±10.4	0.123
**Hek293t**	Mastic oil	0.5%	100%±0.0	1.00
		0.05%	100%±0.0	1.00
		0.005%	100%±0.0	1.00
	Sesame oil	0.5%	100%±0.0	1.00
		0.05%	100%±0.0	1.00
		0.005%	100%±0.0	1.00
	5-FU	250µg/ml	6.7±0.9%	0.008*
		100µg/ml	7.9±1.2%	0.024*
		10µg/ml	51.0±12.6%	0.234
		1µg/ml	81.3±12.5%	0.542

Compare to control group (full viability 100%).* P<0.05 (Student’s t-test).

## References

[1] Papalois A, Gioxari A, Kaliora AC, Lymperopoulou A, Agrogiannis G, Papada E (2012). Chios mastic fractions in experimental colitis: implication of the nuclear factor kappaB pathway in cultured HT29 cells. J med food.

[2] Mahmoudi M, Ebrahimzadeh MA, Nabavi SF, Hafezi S, Nabavi SM, Eslami S (2010). Antiinflammatory and antioxidant activities of gum mastic. Eur Rev Med Pharmacol Sci.

[3] Triantafyllou A, Bikineyeva A, Dikalova A, Nazarewicz R, Lerakis S, Dikalov S (2011). Anti-inflammatory activity of chios mastic gum is associated with inhibition of tnf-alpha induced oxidative stress. Nutr J.

[4] Paraschos S, Mitakou S, Skaltsounis AL (2012). Chios gum mastic: A review of its biological activities. Curr Med Chem.

[5] Dabos KJ, Sfika E, Vlatta LJ, Frantzi D, Amygdalos GI, Giannikopoulos G (2010). Is chios mastic gum effective in the treatment of functional dyspepsia? A prospective randomised double-blind placebo controlled trial. J Ethnopharmacol.

[6] Dabos KJ, Sfika E, Vlatta LJ, Giannikopoulos G (2010). The effect of mastic gum on helicobacter pylori: A randomized pilot study. Phytomedicine.

[7] Djerrou Z, Maameri Z, Hamdi-Pacha Y, Serakta M, Riachi F, Djaalab H (2010). Effect of virgin fatty oil of pistacia lentiscus on experimental burn wound’s healing in rabbits. Afr J Tradit Complement Altern Med.

[8] Djerrou Z, Hamdi-Pacha Y, Belkhiri AM, Djaalab H, Riachi F, Serakta M (2011). Evaluation of Pistacia Lentiscus Fatty Oil Effects on Glycemic Index, Liver Functions and Kidney Functions of New Zealand Rabbits. Afr J Tradit Complement Altern Med.

[9] Rahman HS (2018). Phytochemical analysis and antioxidant and anticancer activities of mastic gum resin from Pistacia atlantica subspecies kurdica. Onco Targets Ther.

[10] Leduc V Moneret-Vautrin DA, Tzen JTC, Morisset M, Guerin L, Kanny G (2006). Identification of oleosins as major allergens in sesame seed allergic patients. Allergy.

[11] Dan Lv, Chang-Qing Zhu, Li Liu (2015). Sesamin ameliorates oxidative liver injury induced by carbon tetrachloride in rat. Int J Clin Exp Pathol.

[12] Salehi Gh. Gharabadin Salehi. In:amale Saleh. First ed. Shiraz, Iran, Shiraz university of medical sciences: Traditional medicine and history of medicine research center,Chogan Publications; 2012: 221.[In persian].

[13] Ostovan M, Fazljou SMB, Khazraei H, Araj Khodaei M, Torbati M (2020). The Anti-Inflammatory Effect of Pistacia Lentiscus in a Rat Model of Colitis. J Inflamm Res.

[14] Kang JS, Wanibuchi H, Salim EI, Kinoshita A, Fukushima S (2007). Evaluation of the toxicity of mastic gum with 13 weeks dietary administration to f344 rats. Food Chem Toxicol.

[15] Janakat S, Al-Merie H (2002). Evaluation of hepatoprotective effect of Pistacia lentiscus, Phillyrea latifolia and Nicotiana glauca. J Ethnopharmacol.

[16] Maarouf T, Abdennour C, Houaine N (2008). Influence of Pistacia lentiscus oil on serum biochemical parameters of domestic rabbit Oryctolagus cuniculus in mercury induced toxicity. Eur J Sci Res.

[17] Azab Elsayed Azab (2014). Hepatoprotective effect of sesame oil against lead induced liver damage in albino mice: Histological and biochemical studies. American Journal of BioScience.

[18] Hsu DZ, Chen KT, Lin TH, Li YH, Liu MY (2007). Sesame oil attenuates Cisplatin-induced hepatic and renal injuries by inhibiting nitric oxide-associated lipid peroxidation in mice. Shock.

[19] Hassanzadeh-Taheri M, Hassanzadeh-Taheri M, Jahani F, Erfanian Z, Moodi H, Hosseini M (2019). The impact of long-term consumption of diets enriched with olive, cottonseed or sesame oils on kidney morphology: A stereological study. An Acad Bras Cienc.

[20] Soliman MM, Attia HF, El-Ella GA (2015). Genetic and histopathological alterations induced by cypermethrin in rat kidney and liver: Protection by sesame oil. Int J Immunopathol Pharmacol.

[21] Cheah KY, Howarth GS, Putnam Bastian SE (2014). Grape Seed Extract Dose-Responsively Decreases Disease Severity in a Rat Model of Mucositis; Concomitantly Enhancing Chemotherapeutic Effectiveness in Colon Cancer Cells. PLoS One.

[22] Attouba S, Karam ShM, Nemmar A, Arafata Kh, John A, Al-Dhaheri W (2014). Short-Term Effects of Oral Administration of Pistacia Lentiscus Oil on Tissue-Specific Toxicity and Drug Metabolizing Enzymes in Mice. Cell Physiol Biochem.

[23] Doi K, Wei M, Kitano M, Uematsu N, Inoue M, Wanibuchi H (2009). Enhancement of preneoplastic lesion yield by chios mastic gum in a rat liver medium-term carcinogenesis bioassay. Toxicol Appl Pharmacol.

